# Temperature-Compensated Clock Skew Adjustment

**DOI:** 10.3390/s130810981

**Published:** 2013-08-20

**Authors:** Jose María Castillo-Secilla, Jose Manuel Palomares, Joaquín Olivares

**Affiliations:** Computer Architecture, Electronics and E.T., University of Córdoba, Córdoba 14071, Spain; E-Mails: jmpalomares@uco.es (J.M.P.); olivares@uco.es (J.O.)

**Keywords:** clock skew, WSN, synchronization, temperature, oscillators, tuning-fork, FTSP, AT-FTSP, A2T-FTSP

## Abstract

This work analyzes several drift compensation mechanisms in wireless sensor networks (WSN). Temperature is an environmental factor that greatly affects oscillators shipped in every WSN mote. This behavior creates the need of improving drift compensation mechanisms in synchronization protocols. Using the Flooding Time Synchronization Protocol (FTSP), this work demonstrates that crystal oscillators are affected by temperature variations. Thus, the influence of temperature provokes a low performance of FTSP in changing conditions of temperature. This article proposes an innovative correction factor that minimizes the impact of temperature in the clock skew. By means of this factor, two new mechanisms are proposed in this paper: the Adjusted Temperature (AT) and the Advanced Adjusted Temperature (A2T). These mechanisms have been combined with FTSP to produce AT-FTSP and A2T-FTSP Both have been tested in a network of TelosB motes running TinyOS. Results show that both AT-FTSP and A2T-FTSP improve the average synchronization errors compared to FTSP and other temperature-compensated protocols (Environment-Aware Clock Skew Estimation and Synchronization for WSN (EACS) and Temperature Compensated Time Synchronization (TCTS)).

## 1. Introduction

Time synchronization is critical in most networks, especially in WSNs [1]. Clock accuracy in these types of networks is difficult to achieve, due to energy and processing constraints in the motes. Synchronization protocols [2,3] allow the application of several different policies, such as sensor data fusion, coordinated actuation between nodes and power-efficiency.

The work of Vig [4] demonstrates that the “characteristics of crystal units are determined primarily by the angles of cut of the crystal plates with respect to the crystallographic axes of quartz” and “a temperature variation can also cause a frequency change due to the energy dissipation in the active area of the resonator”. More recently, Sugihara *et al.* [5] demonstrate how the temperature variations affect the TelosB oscillator (CMR200T). Using a set of motes with the above mentioned oscillator, this work demonstrates that the synchronization error of FTSP increases, due to temperature variations. A more accurate clock skew is obtained when temperature variations are included in synchronization protocols.

As is described in Section 3.1, there are several types of oscillators. The common Crystal Oscillator (XO) does not compensate for its frequency on temperature, providing a clock stability of ±20 ppm at 25°C (e.g., Citizen CMR200T). On the other hand, the Temperature Compensated Crystal Oscillators (TCXO) are able to manage temperature variations in order to enclose the errors within a certain range. For example, the Kyocera KT3225T TCXO improves the frequency stability to ±5 ppm. However, the enhancement obtained by TCXO requires more complexity, energy consumption and economic costs. For the previously given models, prices are US$3.10@3K for the TCXO and US$0.25@2K for the XO model. For compensating temperature with an XO, it is mandatory to include a temperature sensor, e.g., a thermistor probe with a cost of US$0.06@2K, which sum up to a total of US$0.31@2K. Therefore, as Yang *et al.* [6] state, “even with a TCXO, the clock skew is still non-negligible (e.g., up to ±7.5 ppm)”. Thus, flexible software solutions working on low-cost hardware systems are a strong and feasible research area in order to improve time synchronization protocols in WSNs.

This work proposes an innovative correction factor to obtain a more precise clock skew, based on temperature variations between the global and the local clocks. With this adjustment, two new synchronization approaches for WSNs are proposed: the Adjusted Temperature FTSP (AT-FTSP) and the Advanced Adjusted Temperature FTSP (A2T-FTSP). Experiments have been carried out in a network of TelosB (Rev.b) motes running TinyOS. Both AT-FTSP and A2T-FTSP improve the average synchronization error with respect to FTSP under temperature variation scenarios.

## 2. Synchronization Protocols in WSN

Synchronization requirements change depending on the final application. Nevertheless, all synchronization mechanisms require the exchange of a minimal amount of messages between nodes. The number of messages is determined by the synchronization protocol used. The quality of the synchronization is measured using the *synchronization error* concept, which shows the differences between the local clock of the nodes compared to a global clock.

### 2.1. Common Synchronization Protocols in WSN

In the last 10 years, many works have been published in the WSN area. Most time synchronization approaches in the scientific literature can be grouped into two main fields: The first group finds its foundations in obtaining the synchronization through the acquisition and the processing of data [7] to estimate the delays using mathematical models. The second group obtains the synchronization by analyzing the delay sources and removing them from the communication in the root node [8]. Both approaches have reached several advances in synchronization protocols [2,3,9–11].

Elson *et al.* proposed the Reference Broadcast Synchronization Protocol [9] (RBS). The beacon frames sent by the reference node do not contain any time information. These frames are used by the nodes to determine the arrival time and, thus, to compare their local clocks using message transfer.

With the goal of improving the RBS protocol, Ganeriwall *et al.* proposed the Timing-sync Protocol for Sensor Networks (TSPN) [10]. It uses a hierarchical topology to create a network with N levels, all of them synchronized with the root node. Two phases are applied to obtain the complete synchronization: the level discovery phase and the synchronization phase. In the level discovery phase, the topology and the levels of the network are created. The synchronization phase is responsible for carrying out the message exchanging between levels to achieve the synchronization in the whole network using MACtime-stamping. The major problem of this approach is in the message exchange. When the network has a large number of nodes, the energy consumption due to send and receive packets is very high.

The Flooding Time Synchronization Protocol (FTSP) was proposed by Maróti *et al.* [3]. It is based in the concept of flooding (broadcasting) packets in WSNs. Each broadcast packet contains synchronization information about the root node of the network. It sends broadcast packets periodically with its local time (which is considered to be the global time of the network). When the broadcast message arrives at its destination, the arrival time is stored using MAC time-stamping. Using the retrieved information and the local MAC time-stamping, a reference point is obtained. It contains information about the global time and the local time of the receiving node. When a certain number of reference points is reached, the nodes can obtain both the offset and the clock skew with high accuracy using linear regression.

Besides the above mentioned mechanisms, a great amount of protocols have been proposed in the literature to synchronize a WSN as, for example, the Reliable Slotted Broadcast Protocol (RBSP), which is based on the concept of broadcast time slots.

### 2.2. Temperature-Based Synchronization Protocols in WSNs

The use of temperature in WSN synchronization protocols is a field of study with high relevance [5,6,12]. Since the publication of RBS, TPSN and FTSP, several temperature-based synchronization protocols have been proposed to improve the performance of the synchronization process. Among these works, two approaches can be highlighted: Temperature Compensated Time Synchronization (TCTS) and the Environment-Aware Clock Skew Estimation and Synchronization for WSNs (EACS).

Schmid *et al.* [12] propose TCTS. It takes into account the temperature sensor built in the motes, and it calibrates the local oscillator by removing the effects due to temperature changes. This approach has a process known as *frequency error estimation*, which determines the clock skew of the local oscillator related to the reference node of the network for every given measured temperature. The authors assume that temperature remains constant for every clock skew estimation. After obtaining the clock skew for a pair of nodes in a given temperature, a new clock skew with a new temperature value is then computed. When the complete range of temperatures has been explored, TCTS begins with the synchronization phase. If a certain temperature is not computed, the calibration process restarts again to include that new skew information. Thus, the node carries out a realistic adjustment of the frequency. Once the relationship between the frequency error and the obtained temperature is learned, the node increases the time between synchronization intervals. This allows an improvement of the synchronization period, minimizing the number of synchronization periods and, thus, optimizing the energy consumption. Schmid *et al.* provide simulation results during a 96 h period. For the first 24 h, TCTS is in the calibration mode. At the end of the simulation, 95% of the errors lie within ±4 tics of a 32 khz oscillator. Results show a clock stability of <0.07 ppm for a 16 h test with no resynchronization.

EACS was proposed by Yang *et al.* [6]. Taking into account temperature variations, Yang *et al.* describe a highly accurate clock skew estimation. The authors propose an additional information-aided multi-model Kalman filter (AMKF) algorithm to dynamically compensate for the clock skew. The great benefit of EACS is the possibility of using it as a component of any conventional WSN synchronization protocol based on the clock skew estimation. This allows the update of the local clock with local information before the clock resynchronization is carried out, which improves the lifetime of the mote by increasing the synchronization period. The EACS algorithm builds up an initial table of each node of the network based on the correlation between temperature and clock skew. A Kalman filtering is applied to obtain the theoretical relationship between those components. This procedure has very large computational requirements, which does not make it a suitable method for low cost WSN. Simulation results show that the error is always below 2 ms for the whole 8, 000-s simulation. Experimental results with a Mica2 testbed provide an error of 8 ms for a 7,200-s test duration. The authors do not provide any measure of frequency stability.

The above mentioned proposals improve the behavior of protocols as, for example, FTSP, under temperature variations. However, both approaches (i) have high computational costs and energy consumption, (ii) require a large amount of memory per node to store the relationship between temperature and clock skew, (iii) cannot manage new temperatures without a recalibration process and (iv) are constrained to always use the same reference node. Any change in these restrictions triggers the need for a new calibration.

## 3. Frequency Variations in Tuning-Fork Oscillators

Nowadays, many electronic devices are controlled by periodical signals. These signals are produced by electronic components, known as oscillators. They are used in a great range of devices, e.g., digital clocks, smartphones, electrical appliances, computers, vehicles, airplanes, *etc*.

The utilization of oscillators involves synchronization problems that have to be solved in order to optimize the performance of electronic devices. The oscillation frequency may vary due to different reasons. This fact produces a variation between the real oscillation frequency and the nominal frequency (which is the frequency given by the manufacturer). The above mentioned variation is usually defined as the *clock skew or clock drift*.

In nature, all the elements have a resonance frequency that allows their atoms to have an oscillatory movement. Several elements can be found in nature for creating a periodical signal, but among all of them, quartz crystals are the most used ones because of their resonance capability with a fixed frequency. The oscillation frequency is mainly due to the cut of the crystal, the temperature and the voltage applied.

A quartz crystal can be cut at different angles. However, the most commonly cut is the right-angled one. Tuning fork oscillators are an example of a right-angled cut. This kind of oscillator is commonly used in most WSN motes, because of its good relation between accuracy and low cost.

### 3.1. Oscillator Categories

The resonance frequency of a crystal unit can vary with temperature. Depending on the method of dealing with the frequency vs. temperature characteristic, Vig [4] classifies crystal oscillators in four categories: Crystal Oscillator (XO), Temperature-Compensated Crystal Oscillator (TCXO), Oven-Controlled Crystal Oscillator (OCXO) and Microcomputer-Compensated Crystal Oscillator (MCXO).

Tuning-fork oscillators, which are a particular case of Crystal Oscillators (XO), are the most commonly used ones in WSNs. However, they are very sensitive to temperature variations. Their clock stability is about ±20 ppm. This kind of oscillator has a quadratic dependency on temperature, which enables the application of a correction factor to improve the clock accuracy. Consequently, it is possible to reduce temperature effects by means of software mechanisms, making them a perfect solution for low-cost WSN motes.

TCXO and OCXO are less used in WSNs, due to their economic costs, footprint and energy consumption. TCXO crystal units use the information provided by an internal thermistor to generate a correction voltage that is applied to a voltage-variable reactance in the crystal network. The reactance variations produce frequency changes proportional to the temperature changes. In TCXO, the clock stability is in the range of 0.5–5 ppm. Their internal components to compensate the frequency with temperature raise their price to about US$1–100 [13]. In OCXO, the crystal unit and other temperature-sensitive components of the oscillator circuit are maintained at a constant temperature inside an oven, providing a clock stability of 0.001–1 ppm. For this purpose, a very precise encapsulation and isolation is required, which raises their price up to US$ 200–2, 000 [13].

Finally, there are certain microcomputer-controlled oscillators available with “self-temperature sensing”, which compensates for temperature variations in the frequency. These oscillators are known as MCXO (Microcomputer-Compensated Crystal Oscillator), with a clock stability of 0.05–2 ppm. The use of a microcomputer increases their size and economic costs (about US$ < 1, 000 [4]), making them inappropriate for their use in low-cost WSNs.

## 4. Influence of Temperature in Clock Skew

Castillo-Secilla *et al.* [14] demonstrate that temperature variations affect the clock skew estimation in synchronization protocols. The following paragraphs show the above mentioned behavior on FTSP.

Using a star-based WSN topology with one root and four standard motes, experiments have demonstrated the influence of temperature variations in the clock skew of a tuning-fork oscillator. The root mote is responsible for the synchronization of all other motes. Once the experiment has started and all the motes are synchronized, a base station receives the local time of each mote in order to generate statistical data about the synchronization error.

In the work of Maróti *et al.* [3], the topology [15] was forced with software mechanisms. In this work, the same configuration to create the topology has been followed. By using the above mentioned topology, a good signal coverage between all the motes and the base station is ensured.

The root mote sends synchronization beacons cyclically, with a 5 s period. The base station broadcasts another beacon to obtain the local time of each mote in a 1 s frequency rate. The linear regression is computed with three elements to obtain a good temporal proximity between data.

**Figure 1.**
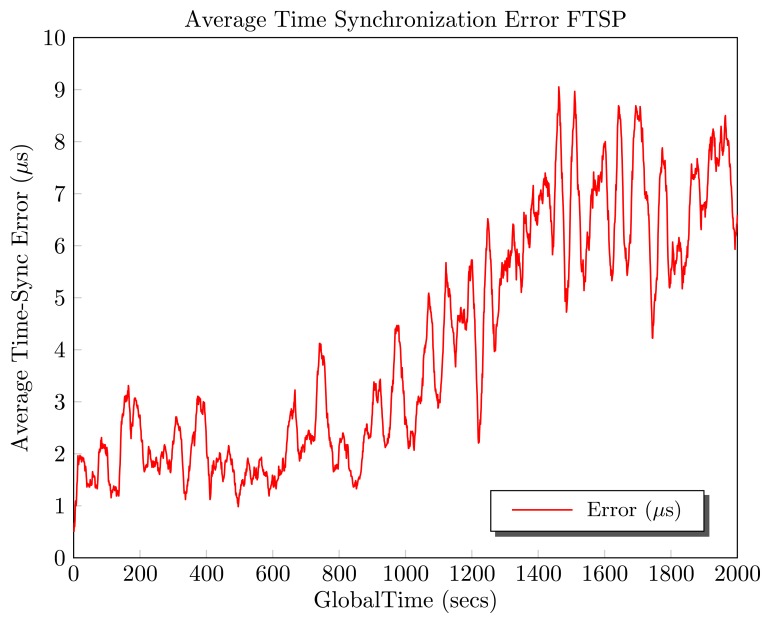
Temperature influence in Flooding Time Synchronization Protocol (FTSP).

The experiment has a duration of 0.5 h, obtaining the following results:

- Between time 0 s and 800 s, the temperature was fixed for all the network. For this period, the average synchronization error was about 1.90 *μs*.
- At 800 s, a temperature variation of 15°C is provoked in just one of the motes of the network. As can be observed in Figure 1, the average synchronization error starts to increase, until instant 2, 000 s.
- The test ends with an average synchronization error of 4.10 *μs*.
- These results show a decrease of the performance of about 215.78%.

The obtained results show a clear influence of temperature in the synchronization error. This is because of the change in the frequency of the oscillator due to temperature variations. The frequency change affects the clock skew estimation, and thus, the average synchronization error increases. These results enforce the postulates of Vig [4], who stated that “temperature variations change the frequency of oscillation”. Therefore, it is necessary to improve the clock skew estimation, taking into account the temperature variations.

## 5. Proposed Methods Based on FTSP

### 5.1. System Oscillator

The motes used in this work have been TelosB (Rev. b) [16]. These motes use a Citizen CMR200T oscillator working at 32.768 kHZ and an internal digitally controlled oscillator (DCO) operating at 1 MHZ. The DCO may be turned on and off. When the DCO is off, the microcontroller (MSP430F1611) operates an external 32.768 kHZ watch crystal. Although the DCO frequency changes with voltage and temperature, it may be calibrated using the external oscillator [16].

Thanks to the work of Maróti and Sallai [17,18], an accuracy of microseconds can be achieved by using a timer linked to the internal DCO. This clock is calibrated by means of the frequency changes occurring in the external 32.768 kHZ oscillator.

**Figure 2.**
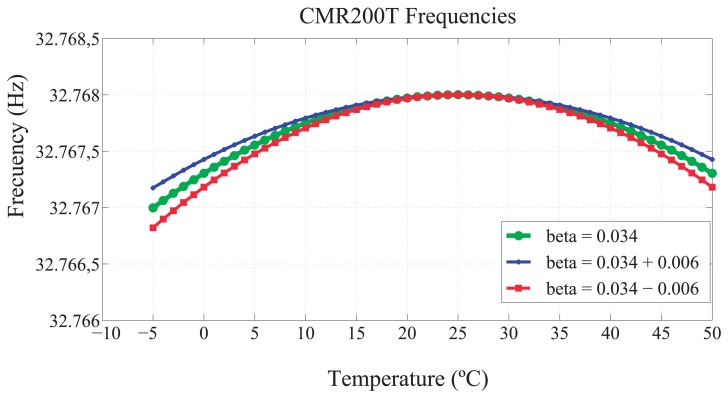
Clock skew in Citizen CMR200T

That external oscillator is classified within the tuning-fork [4] family. The tuning-fork oscillators are characterized by a quadratic relationship between the frequency and the temperature. This relationship is shown in Equation (1) and Figure 2.

(1) $$f=f_{0}(1+\beta {\left(T-T0\right)}^{2})$$

where *f*_0_ is the nominal frequency, *β* is a constant, known as the temperature coefficient, *T* is the ambient temperature and T_0_ is the reference temperature (25°C). In real scenarios, the actual frequency (*f*′_0_) is slightly different when compared to *f*_0_ for a given temperature, *T*_0_. This deviation is known as frequency tolerance. In the CMR200T oscillator, f_0_ = 32,768 Hz, *β* = −0.034 ± 0.006 ppm, and the frequency tolerance is about ±20 ppm. This working interval is mainly due to the effects of the cut of the crystal.

This work proposes two new approaches to obtain a more precise clock skew, taking into consideration the temperature variations [19]. Including the actual temperature of the motes in the computations, it is possible to obtain a correction factor that minimizes the effects of the temperature in the oscillators frequency.

The TelosB model includes the Sensirion SHT11 temperature sensor [20], which allows for an accurate measure, and its performance is guaranteed by the manufacturer.

### 5.2. Adjusted Temperature FTSP (AT-FTSP)

The first approach proposed in this work is called *Adjusted Temperature FTSP* (AT-FTSP). Based on Equation (1), it is possible to obtain a correction factor of the clock skew using the temperature of the local mote. For the sake of helping the reader to better understand the mathematical model, Table 1 collects all the parameters used.

(2) $$f_{n}=f_{0}^{n}(1+\beta_{n}{\left(T_{n}-T0\right)}^{2})$$

Working on Equation (2), Equation (3) has been obtained:

(3) $${\text{Skew}}_{\text{AT}}=f_{0}^{n}(1+\beta_{n}{\left(\Delta T_{n}\right)}^{2})$$

By using the correction factor proposed in Equation (3) and the *clock skew* value of FTSP, a new *clock skew* value is obtained:

(4) $$\text{Skew}={\text{Skew}}_{\text{FTSP}}\cdot {\text{Skew}}_{\text{AT}}$$

AT-FTSP is to be used in those environments where the clock of the reference node cannot be . For example, AT-FTSP is the perfect option for a scenario where the temperature sensor of the reference node is broken, miscalibrated or is known to provide Byzantine errors. On the contrary, when the temperature of the reference node is available, A2T-FTSP, which is described below, can be used to incorporate that information in the clock skew computation process.

### 5.3. Advanced Adjusted Temperature FTSP (A2T-FTSP)

The second approach is called *Advanced Adjusted Temperature FTSP* (A2T-FTSP). If the temperature in the root mote is known, a more precise *clock skew* in the local mote can be obtained. The only requirement is to have temperature sensors, both in the root and the local motes.

By using Equation (1), the *clock skew* of the root mote and the one of the local mote can be compared. Thus, the *clock skew* value of the local mote can be improved compared to AT-FTSP and FTSP approaches.

(5) $${\text{Skew}}_{\text{A}2\text{T}}=\frac{f_{n}}{f_{r}}$$

Equations (6) and (7) show the values of the variables of Equation (5).

(6) $$f_{n}=f_{0}^{n}(1+\beta_{n}{\left(T_{n}-T0\right)}^{2})$$

(7) $$f_{r}=f_{0}^{r}(1+\beta_{r}{\left(T_{r}-T0\right)}^{2})$$

Thus, the *clock skew* value due to temperature effects is determined by Equation (8).

(8) $${\text{Skew}}_{\text{AT}}=\frac{f_{0}^{n}(1+\beta_{n}{\left(T_{n}-T0\right)}^{2})}{f_{0}^{r}(1+\beta_{r}{\left(T_{r}-T0\right)}^{2})}$$

As the first approximation and in order to reduce the complexity of the formulation, it is possible to assume that β_n_ ≃ β_r_. Using the clock skew of Equation (8), it is possible to determine a correction factor based on temperature. Using this approach, the average time synchronization error can be reduced.

(9) $${\text{Skew}}_{\text{A}2\text{T}}=\frac{1+\beta_{n}\Delta T_{n}^{2}}{1+\beta_{n}\Delta T_{r}^{2}}$$

(10) $$\text{Skew}={\text{Skew}}_{\text{FTSP}}.{\text{Skew}}_{A2T}$$

Before providing accuracy results, it is possible to analyze both proposed methods from a computational point of view. It can be concluded that AT-FTSP is a less complex method than A2T-FTSP, and therefore, it is more suitable for low computational power nodes. Besides, A2T-FTSP requires one to send the temperature of the reference node in each synchronization packet, reducing the bandwidth of the communication channel. However, experimental results shown in Section 7 permit one to evaluate both methods with analytical data, taking accuracy into account.

## 6. Performance Evaluation

### 6.1. Experimental Set Up

This section describes the different technologies used in this work: WSN protocols, operating system and WSN motes. A small review of these technologies is shown based on different works and WSN surveys.

Generally, a WSN is a group of low-cost and battery-powered motes. A WSN has a great range of applications, *i.e.*, ambient monitoring, medical monitoring, security, among others. These applications can be used in real-time scenarios, and thus, it is necessary to ensure the correct performance of the network through two approaches: by optimizing the lifetime of the WSN and by using WSN synchronization protocols.

Nowadays, the research community has a great range of low cost wireless communication technologies. For this work, *IEEE802.15.4*[21] has been selected, due to its lower power consumption communications compared to other technologies as, for example, Bluetooth or WiFi. This characteristic is very important to improve the lifetime of the network, especially in real-time applications. Besides, this communication protocol has open source implementations and is mainly used by the research community. In this work, the implementation used of the *IEEE 802.15.4* stack is developed for its use with the TinyOS [22] operating system.

As a WSN mote, the TelosB model has been selected. The TelosB platform is equipped with a Texas Instruments 16-bit MSP430 microcontroller with an internal DCO working at 1 MHZ and an external 32 kHZ oscillator mainly used by peripherals, one of which is the Sensirion SHT11 temperature sensor [20]. In order to digitize the temperature, a new TinyOS module with real-time capability has been developed. This module works as an independent layer inside the synchronization protocol stack using events.

#### 6.1.1. FTSP, AT-FTSP and A2T-FTSP Configuration

##### Parameters

Both AT-FTSP and A2T-FTSP are based on the concept of FTSP, and thus, the configurable parameters [3] are basically the same. Table 2 summarizes the parameters that have been selected for the experiments in a single-hop network.

##### Microsecond Accuracy in FTSP Implementation for TinyOS

WSN synchronization protocols are designed to determine a global network clock. In order to achieve this, the local clock of the mote has to be obtained with the lowest accumulated error. Maróti and Ganeriwal [3,10] demonstrated that time-stamping the packets in the MAC layer reduces the uncertainty times, and thus, errors are minimized.

There are several FTSP implementations, but, none of the published ones use the timers of TelosB motes in TinyOS to obtain microsecond accuracy The original version of FTSP for TinyOS only supports millisecond accuracy In this work, the FTSP protocol implementation for TinyOS has been improved. With this improvement, microsecond accuracy has been reached, and the results of AT-FTSP and A2T-FTSP can be compared with FTSP in microseconds. To do so, several tasks have been performed. FTSP implementation for TinyOS is based on IEEE 802.15.4 by using the CC2420 communications stack. The above mentioned stack has been developed to provide support in motes with the Texas Instruments CC2420 radio. By default, the stack for the CC2420 does not support MAC time-stamping with microsecond accuracy, and thus, it is necessary to modify it. The CC2420x stack of TinyOS can be used. This communication stack uses the *rfxlink* as the communication driver. In the TinyOS tree, this library is located in */tinyos/lib/rfxlink*. CC2420xActiveMessageC library has been used for the required data encapsulation, while keeping compatibility with CC2420x stack.

As has been described in Section 5.1, the microsecond accuracy is obtained by using the internal DCO of the MSP430, which controls the timer where the local time is stored. TinyOS is responsible for the dynamic recalibration of the clock of the DCO, adjusting its working frequency (increasing or reducing it) according to the external 32 Khz oscillator frequency.

#### 6.1.2. Topology

The experimental IEEE 802.15.4 network is composed of:

- One central mote to obtain all the synchronization data from the network, with the goal of storing the information in a file to process it with MATLAB.
- One mote the broadcasts beacon frames based on the BEACON_RATE parameter.
- Thirteen motes flashed with FTSP, AT-FTSP or A2-FTSP One of them is the root mote, which keeps the global time of the network. Between the final nodes, only three nodes (randomly selected per test) suffer from the variation of temperature, as can be seen in Section 7.

#### 6.1.3. Data Extraction

In order to ensure the reliability of the extracted information, each configuration of the network has been executed for 3,000 s.

To determine the average synchronization error, the sequence number of each packet has been used. Packets with the same sequence number are grouped to determine the average synchronization error in each group (Equations (11) and (12)). Once the average error for each sequence number has been obtained, the global average synchronization error is determined using Equation (13).

(11) $$\text{AvgBeacon}=\frac{\sum_{i=1}^{\text{nodes}}(\text{GlobalTime}[i])}{\text{nodes}}$$

(12) $$\text{ErrorBeacon}=\frac{\sum_{i=1}^{\text{nodes}}(\text{AvgBeacon}-\text{GlobalTime}[i])}{\text{nodes}}$$

(13) $$\text{ErrorGlobal}=\frac{\sum_{j=1}^{\text{beacons}}(\text{ErrorBeacon}[i])}{\text{beacons}}$$

Other parameters with statistical relevance have been obtained: variance, typical deviation and maximum error.

## 7. Results

This section describes the experiments carried out with temperature variations. These variations demonstrate a better performance of the new approaches compared to FTSP.

Three temperature-based cases of study have been considered for the experiments:

**Low temperature:** Cases in which the temperature range is between 9°C–22°C.
**Intermediate temperature:** In this range, the temperature is near the nominal value of the *CMR200T* oscillator. The temperature range is between 22°C–32°C.
**High temperature:** The last temperature range encloses temperatures between 22°C–40°C.

Several ranges of temperature are used to study the performance and behavior of the proposed approaches. In order to obtain a good relationship between synchronization and energy consumption, a BEACON_RATE of 30 s has been selected. This rate is appropriate to test the effects of temperature in relation to the oscillator frequency. A slower synchronization rate would not clearly show the frequency variations due to temperature, and also, the energy consumption would be highly penalized. The linear regression uses three elements to obtain information about offset and clock skew, using temporally closer values.

### 7.1. Low Temperature: 9°C–22°C

This section shows the results obtained with the configuration parameters as detailed in Section 7. All the proposed methods are affected by a temperature variation in the range of 9°C–22°C.

The tests have been carried out for an elapsed time of 3,000 s. A temperature variation is created at instant 1,000 s, where the temperature has been decreased from 22°C to 9°C.

**Figure 3.**
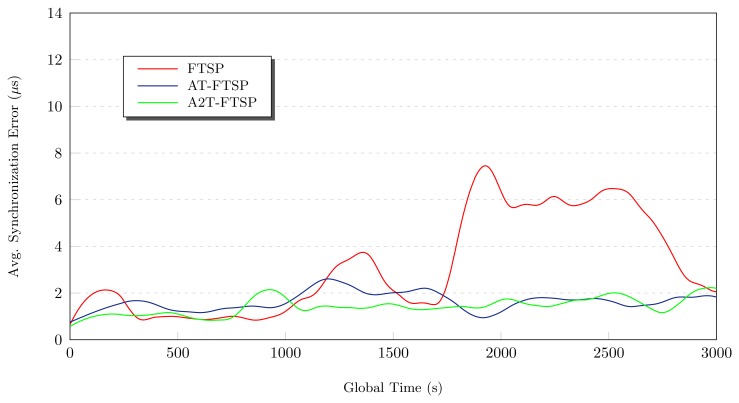
Behavior study: 9°C–22°C.

At the end of this subsection, a linear graphic is shown. This one (see Figure 3) shows the comparative behavior between all the approaches. Also, the statistics results are summarized in Table 3.

#### 7.1.1. FTSP

**Figure 4.**
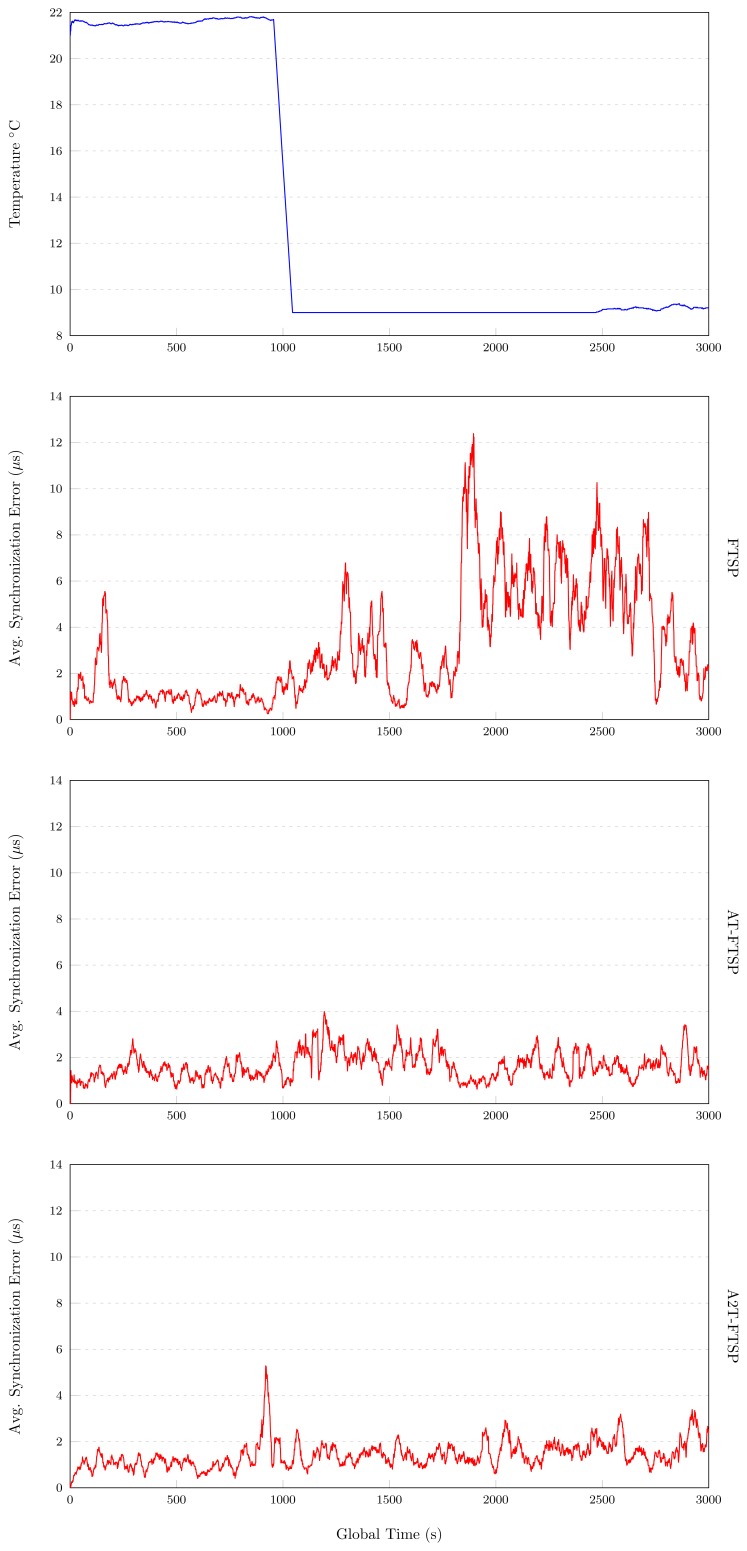
Low temperature results.

Figure 4 shows the results obtained in a low temperature scenario with FTSP. During the first 120 s of the test, a light increase of the average synchronization error is observed. This situation is completely normal because, at the start of any experiment, the linear regression elements are temporally distant between them, and thus, the average synchronization error becomes larger. After the first few execution periods of the test, the average synchronization error improves, until instant 1, 000 s is reached, where a temperature variation is induced. The influence of the temperature is evident, and it affects the average synchronization error, until instant 2,500 s, where it improves, due to the stabilization of the frequency of the oscillator. The test ends with an average synchronization error of 3.181 *μs*.

#### 7.1.2. AT-FTSP

During the first 1,000 s of the test, a good performance of AT-FTSP is observed, with an average synchronization error of 1.1 *μs*. After the temperature change, the average synchronization error increases more slowly compared to FTSP, reaching an average synchronization error of 1.9 *μs*, until time 1, 700 s. Ending that instant, the average synchronization error decreases back to the initial values. This behavior demonstrates a good performance. The final average synchronization error is 1.558 *μs*. The obtained speedup improves in 104.17% the results of FTSP.

#### 7.1.3. A2T-FTSP

The graphical chart of A2T-FTSP (see Figure 4) shows a similar behavior compared to AT-FTSP. During the test, the average synchronization error behavior is good, despite the temperature variation. At instant 1, 000 s, the average synchronization error suffers a small drift, going from 1.1 *μs* to 1.459 *μs*. These results show a clear improvement, obtaining a speedup of 118% over FTSP.

##### Conclusions: Low Temperature

After analyzing the tests with a temperature variation between 22°C and 9°C with three elements in linear regression and a beacon rate of 30 s, it can be concluded that:

- The inclusion of the temperature (see Figure 4) to obtain the clock skew value improves the performance. As a consequence, the average synchronization error decreases.
- Temperature-based methods improve the average synchronization error between 104.17% and 118% compared to FTSP.

### 7.2. Intermediate Temperature: 22°C–32°C

Once research with low temperatures has ended, new tests with intermediate temperature values are carried out. Temperatures in the interval 22°C–32°C have been considered to be intermediate values.

Each test starts by fixing the temperature to 22°C during 1, 000 s. After that, a temperature variation is forced, until the temperature reaches 32°C. This variation creates a modification in the clock skew of the oscillator, due to the change of the frequency, and thus, this affects the performance of the synchronization protocols. The final results are summarize in Table 4.

#### 7.2.1. FTSP

**Figure 5.**
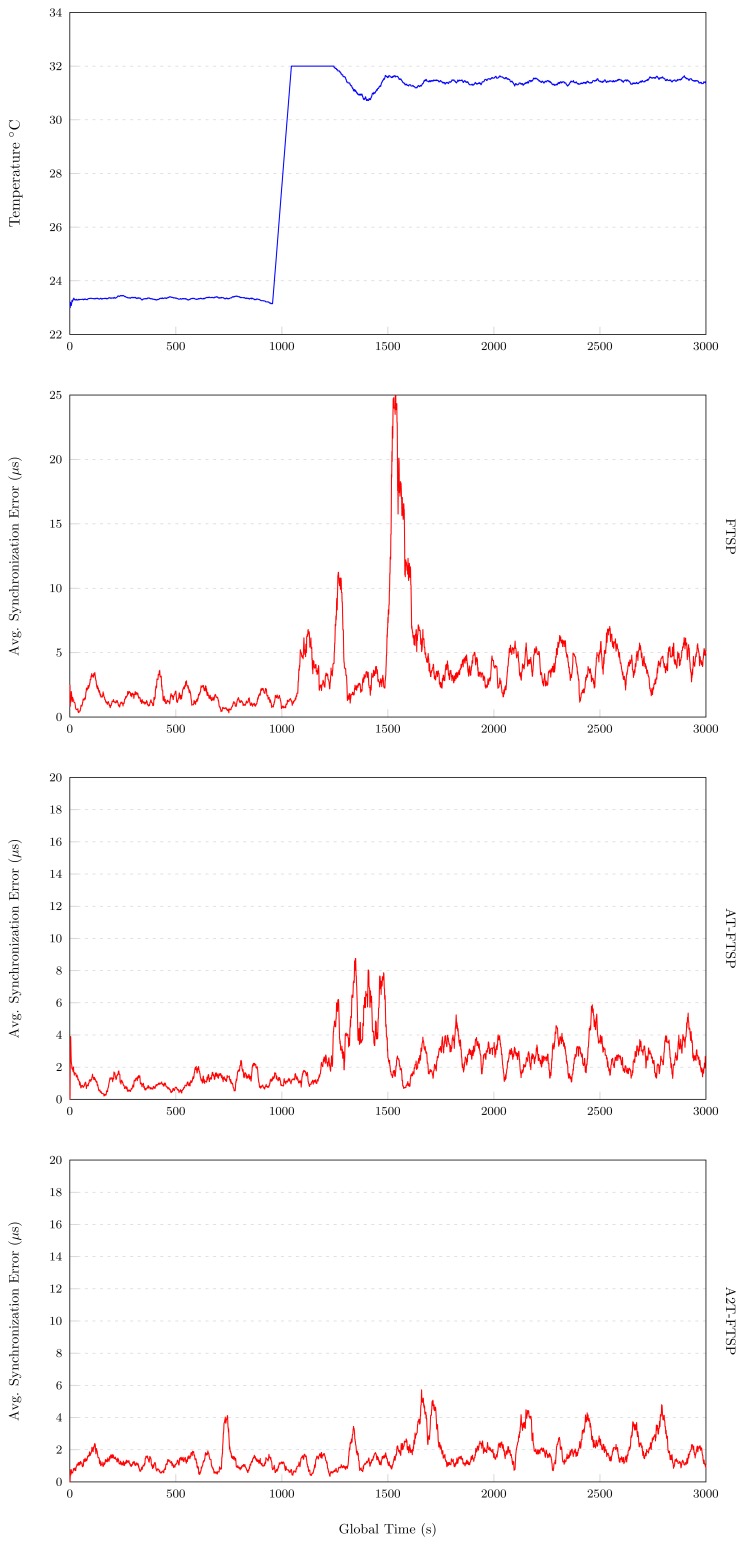
Intermediate temperature results.

In Figure 5, the results of the test executed with FTSP in an environment with a temperature variation between 22°C and 32°C can be observed.

For the first 1, 000 s, a stable behavior can be observed, obtaining an average synchronization error of 1.488 *μs*. A temperature variation is created at instant 1, 000 s. The influence on the synchronization error is evident, and it can be observed that the average synchronization error increases drastically at 1,100 s, 1,250 s and 1,500 s. After instant 1,500 s, the average synchronization error stabilizes to 4.624 *μs*. At the end of the experiment, the average synchronization error is about 3.582 *μs*.

These results show the great influence of temperature in FTSP, and once again, new approaches are required to improve this issue.

#### 7.2.2. AT-FTSP

Figure 5 shows the results of AT-FTSP with a temperature variation between 22°C and 32°C.

Attending to the ordinate axis, a great improvement with respect to FTSP can be observed, both in average synchronization error and behavior. During the first temporal interval (0 s and 1,000 s), an average synchronization error of 1.267 *μs* has been obtained. From instant 1, 000 s until the end of the test, the temperature variation increases the average synchronization error to 1.934 *μs*.

The final average synchronization error is about 2.302 *μs*. This result improves the average synchronization error of FTSP by about 55.6%.

#### 7.2.3. A2T-FTSP

The results obtained with A2T-FTSP can be studied in Figure 5. Once again, the temperature variation oscillates between 22°C and 32°C.

A2T-FTSP has a great performance from the beginning of the test. For the first 1,000 s of the experiment, the average synchronization error is about 1.123 *μs*, with an almost completely constant behavior during all of this period. As is programmed, at instant 1,000 s, a temperature variation is created, going from 22°C to 32°C. At instants 1,200 s and 1,500 s, a slight increase in the average synchronization error is observed. However, once A2T-FTSP reaches instant 1, 500 s in the test, a great improvement in the average synchronization error can be observed, ending the test with an average synchronization error of 1.712 *μs*, which is 109.96% better than FTSP.

##### Conclusions: Intermediate Temperature

**Figure 6.**
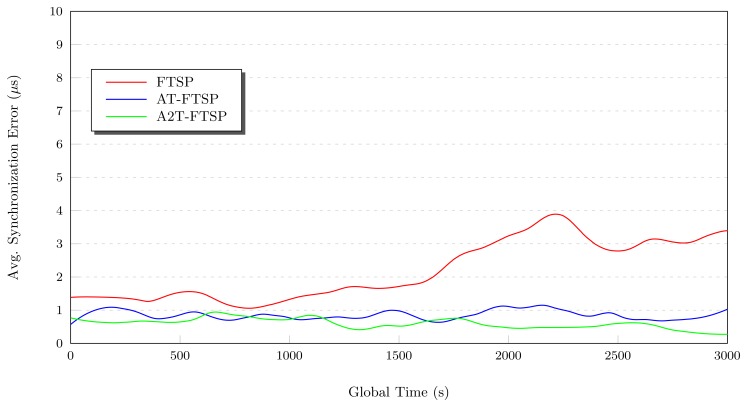
Behavior study: 22°C–32°C.

Analyzing the results with a temperature variation between 22°C and 32°C, it can be concluded that:

- As Figure 6 shows, the use of temperature-based synchronization protocols improves the average synchronization error compared to FTSP under the same conditions.
- The behavior differences between the new proposals are minimal (see Figure 5). This is mainly due to the thermal interval used, which is really close to the nominal frequency of the *CMR200T* oscillator (25°C). As a consequence, the frequency variations are minimal, obtaining similar results between the new methods.
- By using both AT-FTSP and A2T-FTSP, an improvement in the average synchronization error is obtained, ranging from 55.60% to 109.22% compared to FTSP.

### 7.3. High Temperature: 22°C–40°C

The following paragraphs show the experimental results obtained with the parameters described in Section 7. These tests are affected by a temperature variation from 22°C to 40°C. The results of the above mentioned tests are shown in Table 5.

#### 7.3.1. FTSP

**Figure 7.**
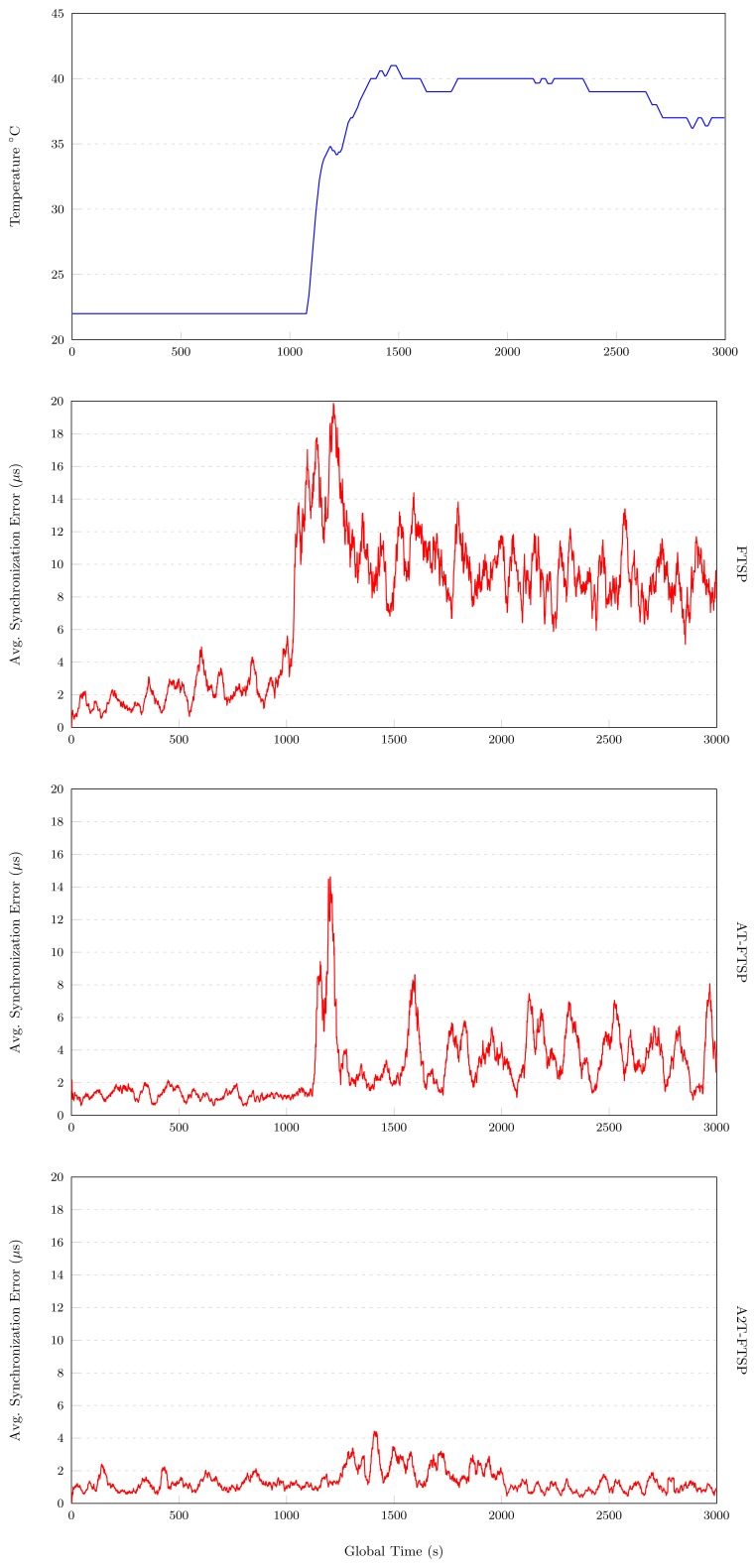
High temperature results.

The results obtained with FTSP (high temperature range) are shown in Figure 7. These results highlight the great influence of temperature in the behavior of FTSP. For the first 1,000 s of the experiment, the temperature is fixed to 22°C, obtaining an average synchronization error of 2.096 *μs*. Once instant 1, 000 s is reached, the temperature is quickly increased to 40°C. In the above-mentioned Figure 7, an average synchronization error of 20 *μs* at instant 1, 300 s is shown. After this peak, the average synchronization error decreases, until it stabilizes to 10 *μs* for the rest of the experiment. The average synchronization error of this test is about 7.432 *μs*.

#### 7.3.2. AT-FTSP

AT-FTSP (see Figure 7) shows a stable behavior during the first 1,000 s of the test, providing an average synchronization error of about 1.266 *μs*.

A temperature variation at instant 1,000 is induced. This change is shown in Figure 7 at instant 1,100 s, where the maximum average synchronization error reaches the value of 15 *μs*. In a short period of time, the proposed clock skew module works to obtain a better synchronization error, minimizing its value to 3.679 *μs* for the second interval (1, 000 *s*–3, 000 *s*).

This test ends with an average synchronization error of 2.685 *μs*, and this means an improvement of 159.40% with respect to FTSP.

#### 7.3.3. A2T-FTSP

Once both FTSP and AT-FTSP have been analyzed, this set of experiments end with the results of A2T-FTSP for high temperature ranges.

For the temporal range without temperature change (0 s–1,000 s), A2T-FTSP has a good behavior and a better average synchronization error (1.1 *μs*). Nevertheless, after applying a temperature variation on the nodes, the average synchronization error increases drastically, producing a peak average synchronization error of 4.25 *μs*. After this situation, A2T-FTSP starts to apply the new clock skew method, decreasing the average synchronization error gradually, until the end of the test, where the error returns to similar values compared to the first temporal interval. The test ends with an average synchronization error of 2.609 *μs*. The improvement with respect to FTSP is clear, amounting to 184.86%.

##### Conclusions: High Temperature

**Figure 8.**
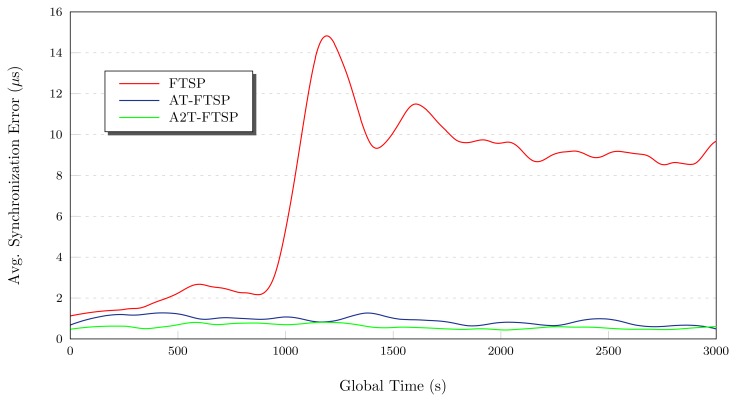
Behavior study: 22°C–40°C.

The tests carried out with FTSP, AT-FTSP and A2T-FTSP (see Figure 8) show an improvement of the new approaches when compared to the original FTSP version. In the following section, the main contributions of these tests are listed:

- High temperatures penalize, to a large extent, the behavior of the synchronization methods. All the tests have demonstrated the above mentioned penalization in the average synchronization error.
- Once again, the use of the temperature-sensitive clock skew module allows one to reduce the average synchronization error by using a basic temperature sensor. Both AT-FTSP and A2T-FTSP improve the behavior and the average synchronization error between 159.40% and 184.86%.

## 8. Comparison to Previous Approaches

This section compares the new proposals of this work with the algorithms described in Section 2.2 (see Table 6 and Table 7). As a reference, the TCTS and EACS algorithms were chosen, because (i) these synchronization protocols take into account temperature variations, (ii) are based on obtaining the clock skew of the nodes to improve the synchronization process and (iii) are designed to be used in WSNs. It is out of the scope of this work to readapt TCTS and EACS algorithms to the experimental TelosB testbed described in this article, due to the complexity of the task. Therefore, only the results provided in the original articles are compared in this work. Nevertheless, the comparison is hard to obtain, because of the experimental setups designed by their respective authors. Those experiments tried to obtain large resynchronization periods instead of looking for decreasing the average error with microsecond accuracy for static synchronization periods.

For the EACS scheme, the authors provided simulated and experimental results. During the whole 8, 000 s simulation, the error was always below 2 ms. On the other hand, for the experimental setup with Mica2 motes, the error was always below 8 ms over the 7, 200 s test duration. The authors established that resynchronization periods for simulation can be prolonged more than 1, 500 s, while the resynchronization period can be enlarged to around 1, 000 s, with a 1 ms resynchronization threshold. Therefore, the frequency stability of this virtual clock with an error of 8 ms in a period of 1, 000 s was 8 ppm.

Schmid *et al.* [12] carried out simulated experiments. Results show that TCTS, on the average, has an average beacon interval of 329 s, and 95% of the errors lie within ±4 tics of a 32 Khz clock (122 *μs*). Taking into account these values, the clock stability is, approximately, 0.37 ppm. However, for very large periods of resynchronization, the authors obtained < 0.07 ppm (4 ms of error in a 16 h period).

On the other hand, the proposed AT/A2T-FTSP methods have obtained similar values in clock stability terms as TCTS for large resynchronization periods. TCTS provided very good results under simulation; however, real-world scenarios include many uncertainties that modify the results from the ideal simulation. Those variations from the simulation results to the real-world experiments have been clearly stated in EACS, where simulation provided an error of 2 ms, while in the experimental setup, that error went up to 8 ms. In this work, all the results are obtained in a real environment and not a simulated one. Nevertheless, the real-world results from AT/A2T are in the same range as the simulation results from TCTS. It is worth noting that neither AT nor A2T require any calibration period, meanwhile TCTS uses 24 h to obtain a good calibration phase.

Besides, as the proposed methods use a 1 MHZ timer compensated for by the 32 KHZ oscillator, microsecond accuracy can be achieved. For the sake of comparison, errors are provided in terms of 1 MHZ clock ticks in Table 6. In that table, average synchronization is used to compute a 1-MHZ tic error. Besides, a maximum error in 95% of periods (the same methodology as described in TCTS) is used to provide another measure of error ticks in a 1 MHZ clock.

These results clearly show the accuracy of the proposed methods and outperforms the previous approaches for the experimental setup described in this work. The simplicity of the proposed methods makes these less computationally demanding than the previous approaches. Besides, the proposed methods do not require any calibration period for temperature compensation. Finally, they are able to deal with dynamic reference node swapping, which was not feasible in previous approaches.

## 9. Conclusions

In this work, two new methods to obtain a more precise clock skew based on temperature for synchronization protocols in WSNs have been proposed. Oscillators are highly influenced by temperature changes. The influence of temperature variations in synchronization protocols has been demonstrated using the Flooding Time Synchronization Protocol (FTSP). The obtained results clearly show the influence of temperature and reveal the necessity of creating new synchronization approaches based on temperature.

Taking into account temperature variations in the clock skew estimation, two new FTSP-based approaches have been developed and tested: AT-FTSP and A2T-FTSP Both approaches use the tuning-fork equation to determine the real frequency of the oscillator according to the ambient temperature. With this information, the clock skew estimation is improved to obtain a more precise value, minimizing the effects due to temperature.

Several tests have been carried out to determine the performance under different temperature variations. The experimental results with the new proposals reveal a great improvement in terms of average synchronization error. The following lines summarize the results of the tests:

- Taking into account the temperature in the computation of the clock skew minimizes the error due to frequency variations. This work has shown the reliability of the new proposed temperature methods for a large range of temperatures (from 9°C to 40°C).
- Using the new temperature-based methods in the range of temperature from 9°C to 22°C permit one to improve the average synchronization error. AT-FTSP provides an average synchronization error of 1.558 *μs* and A2T-FTSP gets about 1.459 *μs*. These results mean an improvement between 104.17% and 118%.
- For the temperature interval, 22°C–32°C, the behavior differences between the new proposals are minimal. This is mainly due to the thermal interval used, which is really close to the nominal frequency of the *CMR200T* oscillator (25°C). As a consequence, the frequency variations are minimal, obtaining similar results between the new methods. For the above-mentioned interval, AT-FTSP and A2T-FTSP improve about 55.60% (2.302 *μs*) and 109.22% (1.706 *μs*) compared to FTSP (3.582 *μs*).
- High temperatures ranges largely penalize the good behavior of the synchronization mechanisms. This is a direct consequence of the big influence of heat. Once again, AT-FTSP and A2T-FTSP improve the average synchronization error, obtaining 159.40% (2.685 *μs*) and 184.86% (1.349 *μs*) with respect to FTSP (7.432 *μs*).

The new methods improve other proposals based on temperature. In order to compare to other temperature-based proposals (EACS and TCTS), results in terms of tics and ppm for <95% of the errors have been obtained. In tics, AT-FTSP and AT2-FTSP offer similar results (±6.74 and ±4.16 tics, respectively) to the TCTS proposal (±4.16). Besides, the results in ppm show a clear improvement of AT-FTSP and AT2-FTSP (0.31 and 0.19 ppm, respectively) *versus* EACS (8 ppm) and TCTS (0.37 ppm). The new methods have fewer computational costs and a better performance, which make them a suitable option to be used in WSNs. Furthermore, it is worth noting that all the obtained results come from real-world experiments and not from simulations.

To summarize the above lines, the main contributions of this article are below:

- The proposed methods can be applied in any synchronization protocol based on the clock skew concept.
- The average synchronization error can be improved, taking into account the temperature. The increase of source code lines is minimal compared to the benefits obtained in terms of synchronization.
- In this work, the used oscillator is the Citizen CMR200T, which has a cost of US$ 0.25@2*K*, and also, the basic thermistor (US$ 0.06@2*K*) is needed to measure the temperature, which makes a total of US$ 0.31. On the other hand, a self-compensated oscillator with temperature (TCXO) can be used to adjust the frequency of the oscillator, for example, the KT3225T oscillator, which is a TCXO model with the same characteristics of CMR200T, but whose cost is about US$ 3.10@3*K*. With the proposed methods, the hardware cost of the motes can be reduced by simply using software mechanism to compensate for the drift of the frequency.
- The basic mechanism (AT) is suitable for environments where the temperature of the reference node is not available (e.g., with faulty temperature sensors). It requires low computational power. Besides, it uses less bandwidth, as no temperature is sent in the synchronization packets.
- The advanced adjustment mechanism (A2T) takes into account both the local temperature and the reference node temperature. It provides better accuracy than the AT method, although it has higher computational requirements and a higher bandwidth usage.

## 10. Future Work

This work will be improved by testing the proposed methods in multi-hop scenarios and/or other time synchronization protocols. It is thought that they will behave properly, because the proposed mechanisms do not depend on the routing protocol, although further testing is required. Lenzen *et al.* [23] have demonstrated that FTSP can be outperformed by their PulseSync algorithm. Thus, it would be interesting to check it with AT/A2T mechanisms.

New experiments are planned. These new experiments will use very large synchronization periods in order to compare these methods under the same scenarios as the ones used in TCTS and EACS.

Finally, these mechanisms are to be designed in simulation platforms, like OMNeT++, which is one of the most commonly used simulators for WSNs. This will allow much more theoretical experiments during longer periods of time.

## Figures and Tables

**Table 1.** Variables in the mathematical model.

**Variable**	**Description**
${\text{Skew}}_{\text{n}}^{\text{T}}$	Correction factor based on temperature.
Skew	Clock skew obtained empirically.
f_n_	Real frequency in node N.
f_r_	Real frequency in root node.
${\text{f}}_{0}^{\text{n}}$	Nominal frequency in node N.
${\text{f}}_{0}^{\text{r}}$	Nominal frequency in root node.
*β*_n_	Temperature coefficient in node N.
*β*_r_	Temperature coefficient in root node.
T_n_	Temperature in node N.
T_r_	Temperature in root node.
T_0_	Nominal temperature (25°C).

**Table 2.** Parameters used in FTSP, AT (Adjusted Temperature)-FTSP and A2T (Advanced Adjusted Temperature)-FTSP.

**Parameter**	**Value**
Beacon Rate	30 s
Max Entries	3 elements
Root Timeout	5 periods
Ignore Root Msg	3 periods
Entry Valid Limit	3 elements
Entry Throwout Limit	500 *μs*

**Table 3.** Results with temperature variation: 9°C–22°C.

Parameter	FTSP	AT-FTSP	A2T-FTSP
Error	3.181 *μs*	1.558 *μs*	1.459 *μs*
Standard Deviation	5.829 *μs*	2.320 *μs*	2.098 *μs*
Maximum error	50 *μs*	27.5 *μs*	23 *μs*
< 95% Error	15.5 *μs*	6.44 *μs*	5.5 *μs*

**Table 4.** Results with temperature variation: 22°C–32°C.

Parameter	FTSP	AT-FTSP	A2T-FTSP
Error	3.582 *μs*	2.302 *μs*	1.706 *μs*
Standard Deviation	6.156 *μs*	3.850 *μs*	2.522 *μs*
Maximum Error	87.500 *μs*	36.500 *μs*	16.222 *μs*
< 95% Error	13.3 *μs*	10 *μs*	7 *μs*

**Table 5.** Results with temperature variation: 22°C–40°C.

**Parameter**	**FTSP**	**AT-FTSP**	**A2T-FTSP**
Error	7.432 *μs*	2.685 *μs*	1.349 *μs*
Standard Deviation	8.757 *μs*	4.941 *μs*	2.064 *μs*
Maximum Error	44.5 *μs*	35.5 *μs*	22.5 *μs*
< 95% Error	22.5 *μs*	12 *μs*	5.5 *μs*

**Table 6.** General results.

**Temperature Range**	**Protocol**	**Average Error tics**	**Avg. ppm**	**<95%Error tics**	**<95%ppm**
**Low**	AT-FTSP	±0.78 tics	0.05 ppm	±3.22 tics	0.21 ppm
	A2T-FTSP	±0.73 tics	0.04 ppm	±2.75 tics	0.18 ppm

**Intermediate**	AT-FTSP	±1.15 tics	0.07 ppm	±5 tics	0.33 ppm
	A2T-FTSP	±0.85 tics	0.05 ppm	±7 tics	0.23 ppm

**High**	AT-FTSP	±1.34 tics	0.09 ppm	±12 tics	0.4 ppm
	A2T-FTSP	±0.67 tics	0.04 ppm	±2.75 tics	0.18 ppm

**Table 7.** Comparative results. EACS, Environment-Aware Clock Skew Estimation and Synchronization for WSN; TCTS, Temperature Compensated Time Synchronization.

**Protocol**	**<95%Error tics**	**<95%ppm**
EACS	N/A	8 ppm *
TCTS	±4 tics	0.37 ppm
AT-FTSP	±6.74 tics	0.31 ppm
A2T-FTSP	±4.16 tics	0.19 ppm

N/A, Not available data;

* Data with 100% error values.
